# Getting to Know the Inner Self. Exploratory Study of Identity Oriented Psychotrauma Therapy—Experiences and Value From Multiple Perspectives

**DOI:** 10.3389/fpsyt.2021.526399

**Published:** 2021-05-21

**Authors:** Sigrid Stjernswärd

**Affiliations:** Department of Health Sciences, Faculty of Medicine, Lund University, Lund, Sweden

**Keywords:** attachment, grounded theory, psychotrauma, trauma, trauma theory, therapy

## Abstract

Early trauma and failures in attachment attunement can affect future relational patterns, health and well-being. The processing of trauma, especially complex trauma, through adequate interventions may help integrate traumatic experiences, enhance health and quality of life. Despite years of clinical practice with Identity oriented psychotrauma therapy (IoPT), there is a lack of scientific research on the subject.

**Objective:** The study's aim was to explore the experiences and value of IoPT for persons with experiences of IoPT, whether as a therapist, client, representative and/or observer.

**Methods:** The study has an explorative, qualitative design. Data collection through individual in-depth interviews and focus groups with 20 participants and data analysis were inspired by grounded theory.

**Results:** The results showed an exploratory process of self-discovery and self-development, *Getting to know the inner self*, to which all categories were interrelated through their contribution to the process. The findings shed light on the experiences and tangible value of IoPT for the participants from the perspectives of client, therapist, representative and observer. These perspectives were intertwined and illuminated in terms of their contribution to the process.

**Conclusion:** IoPT seems to have transformative potential in terms of a self-exploratory journey from multiple perspectives. The need for effective treatments to enhance health and prevent further ill health in persons affected by complex trauma motivates the exploration of novel treatment approaches and formats to support clients toward health enhancing strategies. Further quantitative and qualitative research is motivated to enhance our understanding of the workings and value of IoPT for self-development, health and quality of life.

## Introduction

### Trauma and Mental Health

Attachment patterns are central in children's development of security and autonomy, laying the ground for future relational patterns (1–3). Attachment and trauma research demonstrate that interpersonal and attachment related trauma, whether due to e.g., abuse or neglect, can increase the child's neurobiological sensitivity (4). In combination with environmental factors (5), this may increase the risk for psychopathology such as anxious conditions (6) or dissociative symptoms (7, 8). Unless attachment trauma is processed, it may lead to the maintenance of originally useful defense strategies, which can be unfavorable long term (9, 10). Although dated, some figures show that secure attachment is found in 58% of adults and teenagers in non-clinical populations, but only in 8% of clinical populations and high-risk groups (11). Up to 40% in clinical populations and 19% in non-clinical populations show a disorganized attachment pattern (12). In non-clinical children populations, ~15% show a disorganized attachment pattern, with up to 25% in groups with low socioeconomic status (13). Repeated traumatic experiences in childhood tend to concur (14) and are associated with long-term mental suffering (15). Depression (16) and dissociation (17) are well-documented consequences of childhood abuse. The prevalence of adults with experiences of childhood abuse is 27–60% in the general population (18, 19) and 94% in clinical populations (20). Exposure to psychotrauma, including interpersonal psychotrauma, is common by midlife adulthood (21, 22) and whether stemming from childhood (23) or adulthood, such exposure can impact health negatively across the lifespan (24, 25).

Exposure to trauma, especially early and repeated exposure that lead to cumulative effects of adverse childhood events, is associated with increased risk factors, risky health behaviors, and mental and somatic conditions that represent leading causes of death (26, 27). Victims of childhood emotional abuse and neglect seem to suffer at least equal immediate and long term effects across multiple domains of functioning as victims of violence and maltreatment, including damaging effects on self and identity (28). Complex trauma exposure in youth can lead to a compromised capacity for self-regulation and interpersonal relatedness, increased risks for additional trauma exposure and cumulative health impairments, including addictive disorders (29). Besides increasing the risk for future psychopathology (30), early trauma can be transmitted across generations (31, 32) unless processed, e.g., through healing relational experiences or therapy (13, 33). Severe stress exposure in parents, even before conception of offspring, is thus a risk factor for psychopathology and other adverse outcomes in offspring, although the mechanisms of transmission have not been clearly elucidated (31). Hypotheses are stress vulnerability related to genetic risk factors, behavioral alterations due to stress-related psychopathology (31), and epigenetic changes in offspring transferred by parents whose biological systems have endured such changes as a response to stress exposure, i.e., “intergenerational transmission” (34).

Catastrophic experiences can lead to PTSD, while relational trauma in form of parent-child attunement failures can produce a sense of helplessness, anxiety, shame, humiliation, and abandonment, when left unrepaired by caregivers (35). Such trauma engenders anger, defenses, and insecure attachment patterns, which can be further activated by additional traumatic experiences (35). It is not the event *per se* but its effects that characterize traumatizing events (36). Whether trauma is impersonal, interpersonal, or attachment related (36), it thus encompasses the event itself and the individual's subjective reaction to it (37). It can induce hyper- and hyporegulation of emotional and physiological arousal (38) and result in splitting of personality, where one parts relives the trauma and the other tries to avoid anything that reminds of it (39, 40). Emotional dysregulation and dissociation hinder the integration of emotional memories with other experiences as the right brain hemisphere disintegrates (41), engendering a modified memory function through suppression (42), fragmentation of memories, or priority of sensory perceptions over events by memory (43). Therapies that activate such memories can help reach and process implicit, unconscious memories. The left hemisphere focuses verbal and conscious information. Emotion-related memories, recognition of non-verbal communication and embodied emotional information have their center in the right hemisphere, as do relational contexts such as psychotherapy and the processing of emotional stimuli and communication (41). Sensorimotor therapies, for instance, address multiple information processing levels (i.e., cognitive, sensorimotor, emotional) in the treatment of trauma (44). Somatic approaches that use the clients' interoceptive, proprioceptive and kinesthetic sensations as therapeutic tools may also be valuable venues to explore as supplements to cognitive and exposure therapies in the treatment of trauma (45). Effective treatment approaches that can help clients deal constructively with the consequences of trauma and injurious prolonged stress are essential to prevent continuous painful, and at times, (self) damaging life trajectories with further psychiatric and somatic health complications (27).

### Trauma Therapy

Both trauma-focused and non-trauma-focused therapies are available for the treatment of PTSD, with the latter focusing symptoms rather than traumatic memories (46, 47). For the treatment of PTSD in adults, there are strong recommendations for the use of Prolonged Exposure (PE), Cognitive Processing Therapy (CPT) and trauma-focused Cognitive Behavioral Therapy (CBT), which all address traumatic memories or thereto associated thoughts/feelings (47). Further recommended treatments include Cognitive Therapy (CT), Eye Movement Desensitization Therapy (EMDR), Brief Eclectic Therapy (ET), Narrative Exposure Therapy (NET) and written exposure (47). Rather than treating symptoms, trauma therapy focuses on untying the problems that the traumatized personality suffers from (48). Trauma therapy can be an effective interpersonal intervention, especially as therapy resistant PTSD cannot heal through medication (48). Some of the therapies targeted at trauma survivors, also in digital formats, seem promising with positive short and long-term effects (49). However, there is no “one size fits all,” individuals respond differently to treatment, and a history of complex trauma may represent complications. Multidisciplinary research on novel therapies and therapeutic formats is thus called for (49–51). The evidence for the treatment of complex trauma and dissociative states is more restricted. Validated interventions may not be specifically designed for persons with complex trauma, nor assessed for such populations with the means representative samples (28). This motivates further research to explore the value and effectiveness of interventions for individuals with experiences of multiple and/or prolonged trauma with subsequent complications, and to assess the individual suitability of such interventions using both quantitative and qualitative research approaches. Clinical recommendations for complex trauma encompass integrated treatment methods, with focus on stabilizing strategies prior to trauma processing (52, 53). Besides regulating strategies, multicomponent interventions can focus traumatic memories and the creation of a trauma narrative, the client-therapist relationship, and dissociative states (28). The exploration of integrative multimodal therapies that allow the processing of information at multiple levels (e.g., cognitive, emotional, sensorimotor), and that sheds light on their potential benefits and risks in relation to trauma treatment is hence motivated.

### Identity Oriented Psychotrauma Therapy

**Figure 1:**
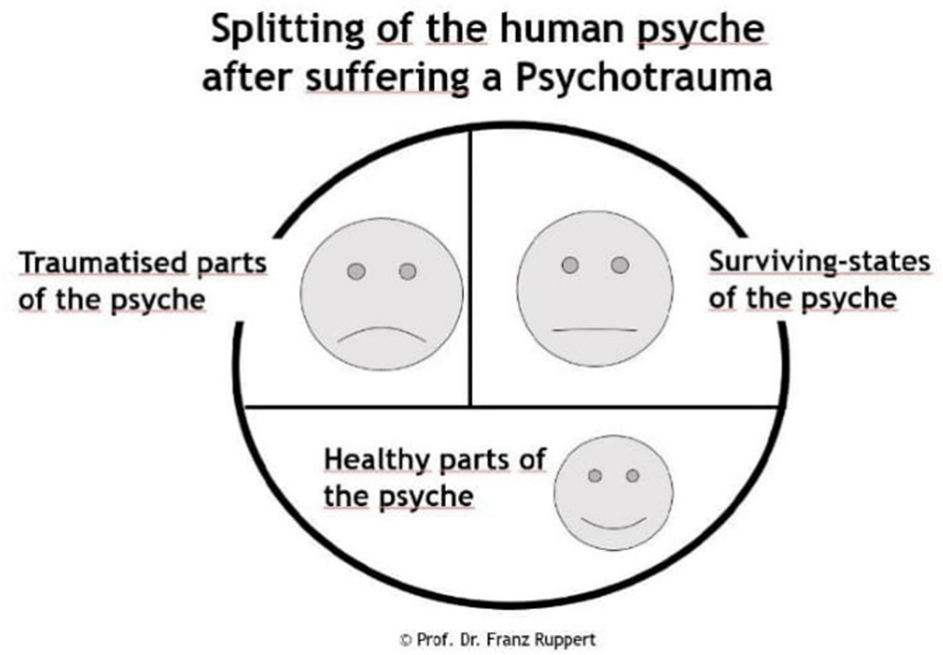
Model of Split of the human psyche after suffering a psychotrauma by Ruppert.

Despite years of clinical practice and an international spread of IoPT training, the area lacks scientific studies, motivating the scientific exploration of the potential benefits and risks associated with IoPT. Psychotrauma theory informs IoPT theory. It focuses on the consequences of psychotrauma on the human psyche, including the subsequent effects on relationships. Ruppert refers to the concepts of human psyche, identity, love, sexuality, the perpetrator-victim dynamic, and works with the so called intention method. In the context of IoPT, focus on trauma refers to the use of *therapeutic/advisory methods and processes* and the *theoretical background* that shapes the method and which is based on attachment and trauma theory (54). As compared to e.g., systemic constellations that focus the client's external family system, IoPT focuses the client's internal system of his/her psychological parts (54). Ruppert (54) pinpoints the importance of staying true to method and theory, as diverging theoretical starting points when using the method may affect the method's beneficial effects, at worst aggravate the client's problem (54). Rather than focusing trauma itself, IoPT focuses the effects of early trauma (e.g., relational trauma) on the individual's identity development, which may include a psychological split of the personality structure as a survival strategy (54–58). Where van der Hart et al. (59) describe a two-part model of trauma consisting of the emotional personality (EP) and the apparently normal personality (ANP), Ruppert's model is three-fold, with the ANP being further divided into the healthy part (HP) and the survival part (SP). Trauma causes fragmentation of the psyche in a healthy part (HP) (healthy structures preceding trauma), a traumatized part (TP) (part of the psyche containing the overwhelming memories and feelings of trauma, which are frozen in the subconscious but may become reactivated), and a survival part (SP) (coping or protective strategies that prevent trauma feelings, memories and sensations to surface) (56) (see Figure 1). IoPT focuses the client's trauma related challenges and survival strategies adopted to handle unaddressed (e.g., attachment) needs, which may complicate relational patterns long-term. The client's therapeutic process can contribute to make visible survival strategies, splits and entanglements; strengthen healthy psychological structures on behalf of survival strategies; and facilitate encounters between healthy psychological parts and traumatized parts (54). Healthy mental structures include a healthy “I,” i.e., a sense of identity with a free will, flexibility and ability to self-regulate. By seeing, recognizing and experiencing these different parts and strategies, a process of change can be activated in the client (54).

As a therapeutic method IoPT includes a, by the client autonomously determined sentence of intention with words/symbols, that represents the issue (s)he wants to address within the IoPT context (example: “*I want to understand my anger.”*) and that the client writes on a whiteboard/flip-chart. Thereby, s(he) makes the intention visible to him/herself and those present in the room. The intention is then processed in a resonance process, through which the different parts of the intention will be resonated with—non-verbally and then verbally, by asking the person that resonates with a specific part to share his/her experiences in the resonance. The process can help the client see and process inner conflicts, entanglements and trauma within a safe therapeutic relation. In group sessions, representatives (also called resonance persons/resonators) chosen by the client resonate with the sentence of intention's respective words/symbols, with one representative per word. The client's internal system of psychological parts will be embodied and shown by the representatives in the client's process, with the representatives embodying different parts of the client's, by trauma, split psyche. The client will get to see, known and maybe unknown, parts of the self through the representatives, and get a chance to integrate these. Once the process is over, the client releases the representatives from their role by saying e.g., “*Thank you for resonating with the word “anger,” now you are Anne again.”*

In one-to-one (OTO) sessions including only the client and the therapist, the therapist and/or markers (e.g., cushions) work as representatives for the sentence of intention's elements in the resonance process. In the first case, the therapist will have a dual role of therapist and resonance person, and will shift between the two. When using markers, the client will move from one marker to the other and thereby experience parts of his/her split psyche as (s)he moves from one marker to the other, or the therapist will resonate consecutively with the separate markers and report his/her experiences to the client. The dynamics between the parts will not be visualized simultaneously as in group sessions. In group sessions (e.g., in treatment or IoPT training groups), persons that are part of the group but not actively participating (e.g., as client, therapist, or representative) are here called observers. In group sessions, led by a therapist and potentially an assisting therapist, participants may be observers only, have an own process as a client, or/and also act as a representative in other clients' processes. Own work through processes as a client is a prerequisite to become a therapist and is included in the training modules. While some therapists mainly work with groups, others work OTO, or in both formats. Therapist and client decide upon the wished for number and frequency of processes, with the client “owning” this decision. IoPT work can thus involve persons acting from different perspectives, i.e., as a client, therapist, representative, and/or observer.

The study's aim was to explore the experiences and value of IoPT in participants with experiences of IoPT, whether as a client, a current therapist or future therapist enrolled in IoPT training, representative and/or observer.

### Ethical Considerations

The study was approved by the Regional Ethical Committee in Lund, Sweden (reg.nr 2018/921). All participants signed a written, informed consent prior to the interviews and agreed on the publication of quotes in the current article.

## Methods

An explorative qualitative approach was chosen for the current study, with a method inspired by grounded theory (GT) for data collection and analysis. GT is appropriate to study unexplored areas and to discover central issues for the participants (60), which are then explained by the emerging substantive theory (61). GT helps deepen the knowledge and understanding of complex processes such as e.g., therapy, without being steered by preconceived theories or hypotheses (60, 62). IoPT can be experienced from multiple perspectives, i.e., as client, therapist, representative, and/or observer. In this explorative study, all perspectives were included to gain an understanding of these perspectives' meaning and potential value for the participants in their experiences of IoPT.

### Participants

Participants were recruited through IoPT institutes' mailing list through which a newsletter including information about the study was emailed. The sample size was thus dependent on the number of interested individuals fulfilling the inclusion criteria that enrolled to the study during the data collection period involving interviews, which was limited to the spring of 2019. Inclusion criteria were: ≥18 years, experience of IoPT from one or multiple perspectives, language (Nordic or English), no ongoing severe mental illness. Twenty informants from the Western world, out of which 16 women and 4 men, participated in the study. Interested participants enrolled to the study by submitting a signed informed consent form to the researcher. The participants' age range was (25–35) (*n* = 1), (36–45) (*n* = 2), (45–55) (*n* = 8), (56–65) (*n* = 4), (66–75) (*n* = 5) with a mean age of 55.3 years. Most participants had a tertiary education (*n* = 18), out of which half included subjects such as psychology, sociology and coaching, or a vocational education (*n* = 2).

Upon enrollment in the study, participants were to choose the perspective(s) they matched and wanted to share their experiences from depending on their preferences and experiences of IoPT from the mentioned perspectives. All participants chose to answer from multiple perspectives, i.e., from the perspective of therapist (currently a therapist *n* = 11 + therapist under training *n* = 4), client (*n* = 19), representative (*n* = 18), and observer (*n* = 14). The No. of resonance processes ever experienced as a client amongst the participants varied from 5 to 100+ (mean = 41). The No. of resonance processes ever held as a therapist (*n* = 11) ranged from 5 to 900 (mean = 252), with the No. of years as practicing therapist (*n* = 9) varying from 1.5 to 9 years (mean = 4). The participants had IoPT training experience of varying levels (*n* = 19, 1 missing): basic training only (*n* = 3), basic + advanced training and/or international advanced training (*n* = 10), advanced and/or international advanced training only (*n* = 4). Two participants (out of 19) did not have any experience of IoPT training as a therapist but only client experience. IoPT training is substantially experiential and includes theory and supervision (IoPT training information: https://www.iopt.no/en/trainings/).

### Data Collection

Participants were free to choose if they wanted to participate in an individual interview or focus group, as this combination of methods has been observed in previous grounded theory studies to enable enriched data (63, 64). Qualitative interviews are motivated to generate in-depth descriptions of the interviewees' world (65), and focus groups to allow the exploration of mutual experiences and identities through interaction, by exploring the participant's mutual interaction and similarities and differences in the participants' answers (66). Data was collected through 16 individual interviews and two focus groups with two informants each over skype/facetime (*n* = 15), phone (*n* = 4), and face-to-face (*n* = 1) (January–April 2019), using the same semi-structured interview guide to cover the different perspectives at stake (see Appendix 1) in both the individual interviews and focus groups.

To cover important aspects of clients' experiences of therapy, questions from the *client perspective* were inspired by the Change Interview (67, 68). It can be combined with open-ended exploratory questions and empathic response to stimulate the informants' elaboration upon their experiences (67). It includes questions on e.g., general experiences of therapy, experienced changes and their attributions, and helpful/hindering/difficult/missing aspects of therapy. Additional questions from the client perspective explored prior experiences of other therapeutic methods (and advantages/disadvantages compared to IoPT) and reasons for choosing IoPT. The *therapist perspective* included questions on e.g., when to (not) use IoPT with clients, type of issues that clients wish to address, processes and mechanisms at work as seen from the therapist perspective, advantages/disadvantages of IoPT, supervision experiences, difficulties experienced as a therapist, and coping strategies to handle potential difficulties related to IoPT work. The *representative perspective* included questions on experiences of being a representative, its value as experienced from the representative perspective, potential difficulties with the role and with separating own material from the client's material. The *observer perspective* included questions on participants' experiences as observers and the experienced value of being an observer of IoPT processes. The interviewer addressed one perspective at a time, using the interview guide's questions from the perspectives chosen by the individual participants in the following order: client perspective, therapist perspective, representative perspective, and observer perspective. Nevertheless, the participants' answers oftentimes covered several perspectives at once, which the participants clearly articulated when that was the case. If not clearly articulated, the researcher asked the informant to clarify which perspective s(he) was speaking from, i.e., if the informant's answer was given from the client, therapist, representative or observer perspective.

Throughout the interviews, the researcher summarized and mirrored back the informants' answers to prompt deepened answers, allowing the participants to confirm, rectify or complete the interviewer's understanding and interpretation of the participants' narratives. As data collection and analysis occur in parallel in GT, the interviewer included questions in the forthcoming interviews based on previous analyses to verify and deepen her understanding of emerging issues, i.e., frequently reoccurring incidents, dimensions of these, and novel aspects brought up in the interviews. The order in which participants were interviewed was based on the principle of theoretical sampling (69), where strategic decisions related to interview order were based on the enrolled participants' age, gender, and country of origin to achieve variation. Repeatedly emerging concepts in the early interviews were further explored in subsequent interviews. A sense of saturation occurred, with the recognition of a core category and related categories as narrated by the participants, and no apparent new data. Hence, another handful of participants that had sent an informed consent was not interviewed, which was also due to time and economic constraints. The interviews, recorded and transcribed verbatim, lasted 58–217 min (mean = 114 min). As several interviews were lengthy, some informants were interviewed at two different occasions.

Notes were taken during and between the educational modules of the International IoPT training (5 modules, 2018–2020), to which the researcher enrolled to deepen her understanding of IoPT. The training provided first-hand and embodied experience of the different perspectives and processes involved, and a theoretical understanding of IoPT theory and method. Literature was read at different stages of the research process. Some literature was kept until the analysis was completed so as not to steer the process (61). A research diary, memos and theoretical notes accompanied the entire research process and were revisited along the whole process, as GT embraces all types of data (61).

### Data Analysis

Data collection and analysis were carried out concurrently and iteratively (70). The transcribed interviews were read several times to get a thorough understanding of their contents. The different perspectives (client, therapist, representative, observer) were explored in the same order during data collection and initially analyzed separately across all interviews. Nonetheless, the different perspectives seemed to be intertwined. Openness to this dynamic was kept in mind throughout the analysis and gradually showed to be a key aspect of the findings. A core category was salient and recurrent across all interviews, pointing toward a process of self-exploration and discovery. All other categories and their properties, which were recurrently occurring in the interviews, were interrelated to the core category either by representing different facets or dimensions of this process or mechanisms contributing to its development. While some categories related to the process and outcome of self-discovery itself, others related to parts of the IoPT method that contributed to this process. The core category, which is interrelated to all other categories, accounts for most of the variation of data. The analysis showed a core category and tentative basic social psychological process of change (71), *Getting to know the inner self*, which represents the participants' main concern.

The analysis proceeded using the constant comparative method, following the different levels of abstraction of open, selective, and theoretical coding, with the latter conceptualizing the categories' interrelations (61). The grounded theory method entails both inductive and deductive elements and is often described as an inductive method with deductive elements. Data collected from a sample is analyzed inductively and steers the next coming sample, which then can be said to be deductively based on the analysis. The transcripts were coded line by line for incidents (e.g., words, sentences, sections) answering the study's aim, i.e., the participants' experiences of IoPT from the included perspectives, and referring to a category or its property. Incidents, which could be words, sentences or sections of text that illuminated the participants' experiences of IoPT (e.g., feeling seen and witnessed, experiencing that the process was led by the client, understanding emotional reactions, and behavioral patterns when seeing them through the representatives, etc.), were coded according to the emerging pattern, i.e., a process of self-discovery. Based on their similarities and differences, coded incidents were gathered into categories, i.e., categories relating to the process of discovery and self-exploration, such as “the subject in the driver seat,” “reclaiming the self,” etc.). Interrelations between the categories and the core category were noted and reflected upon, with the categories representing outcomes or dimensions of the self-discovery process or elements of IoPT contributing to this very process. The transcripts were revisited several times using the emerging pattern, moving back and forth between emerging properties/categories and empirical data (61). The constant comparative method hence implies that incidents are compared to incidents and new incidents are compared to existing categories. Through this process, the central elements, and eventually a theory, will emerge from data and hence be grounded in data. Memos, a research diary and notes from the training modules attended by the researcher were held throughout the whole research process and revisited during the analysis stage, with a documentation of observations, theoretical ideas, and experiences from the training modules; this to facilitate self-reflexivity, bracketing and observation of the development of the researcher's pre-understanding along the research process. The diaries facilitated documentation, representing a paper trail of the researcher's *in vivo* experiences of IoPT processes, expanding understanding of IoPT processes and of how theory and method were enacted in IoPT work.

References to the ternary IoPT model (Figure 1) were recurrent in the participants' narratives and are thus referred to in the findings. As most participants had experiences from all perspectives (client, therapist, representative, observer) and their answers often covered multiple perspectives, the latter are jointly embedded in the results. This choice was also motivated by the apparent inherent value of the participants' experiences of multiple perspectives in their experiences of IoPT processes. Quotes illustrate how the various perspectives relate to the different categories and the researcher's interpretation of data. For enhanced clarity in regards to the quoted perspectives, the interview perspective at stake is denoted within parentheses after each quote: client (C), therapist (T), representative (R), observer (O).

## Results

### Getting to Know the Inner Self

**Figure 2:**
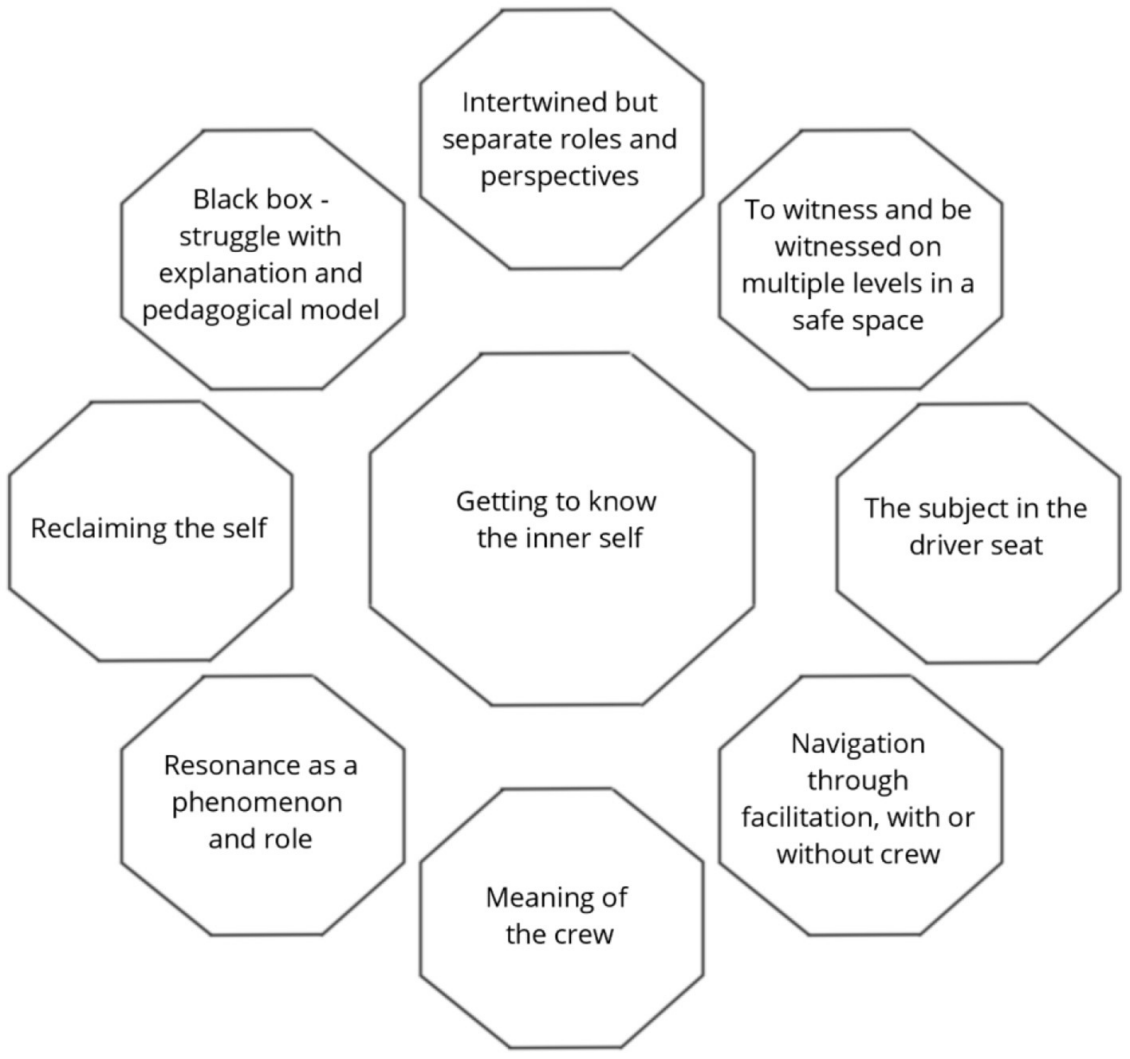
Study findings showing a process of change “Getting to know the inner self” and categories contributing to the named process of self-discovery and inner development.

The core theme, *Getting to know the inner self*, shows that IoPT work permits an exploratory process of self-discovery and self-development from one or multiple perspectives (see Figure 2). The findings show that this process encompasses different facets and mechanisms related to IoPT work that enable this process, as experienced from the perspectives of client, therapist, representative, and observer. These facets and mechanisms from the perspectives voiced by the participants are described in more detail in the categories below. “*The work has enabled me to decide where I want to focus next, in terms of my exploration of myself and my exploration of that other life that I can't remember. // the work has enabled me to decide to put me in the driver seat of where my self-development goes next” (C)*.

### Intertwined but Separate Roles and Perspectives

The exploration of the value and experiences of IoPT from the different experiential perspectives as therapist, client, representative and observer shed light on their intrinsic value. Although the different roles are clearly distinct and revolve around the client's process, they also come through as intimately intertwined and mutually enriching, as illuminated throughout the results. This as participants have experiences from multiple roles, not the least through participation in IoPT training. “*I realise I am partly speaking as a client and partly speaking from a facilitation point of view, I am not sure” (C)*. For a therapist to facilitate IoPT work with clients, (s)he must do own IoPT processes as a client including supervision, thus embodying both roles. Whether as a therapist or a client, the individual may come to act as a representative in other clients' processes, thus experiencing the representative role. In group processes, clients will invite representatives into their processes and in one-to-one sessions, clients may come to resonate with markers and themselves experience shifts between the resonances from the different markers. If not active as a client, therapist or representative, individuals can be observers in e.g., group processes, workshops, or training modules. Observers can also be invited to be representatives in clients' processes. As witnessed by the participants, firsthand experience from all perspectives is not necessary to benefit from IoPT, although a multiplicity of roles may deepen one's experiences, understanding of and trust in the workings of IoPT. It also extends one's understanding of the self and others. “*It's been like that with the training on the whole, it's something that is hundred percent client and hundred percent therapist, where one also learns a method” (T)*.

IoPT has developed over time and offers to work with a client's trauma biography through facilitation, with the subject in the driver seat. Facilitator (or navigator) is a preferred word for therapist as it implies facilitating the client's process rather than offering therapy. It presupposes that the facilitator does his/her own work with the intention method and resonance process, including ongoing supervision focusing the facilitator role—and thus potentially personal issues—and client cases. “*Being a facilitator means you have to keep working on yourself, you have to keep digging deeper and deeper” (T)*. Depending on the process format (group or OTO), either the facilitator and/or representatives and/or markers can embody and act as intermediary in the resonance work, making visible the client's inner entanglements. Resonating with a client's intention can represent an opportunity for self-development also for the representative, as it allows contact with multiple emotions, thoughts and sensations through resonance. “*I can also feel that it starts-up //my own processes when I am a representative for others. I clearly feel that. And when I see recognition in the client that I am resonating with // I feel more secure accepting others when they are representatives for me. // When I share what happens with me, which I know isn't mine, then others do the same when they sense what belongs to me, when I am a client” (C/R)*. The observer perspective permits an overview of the whole process. It is a learning opportunity to study the facilitation process and the client's work, also promoting (self) reflection and an understanding of others. “*When I watch more experienced therapists, I learn how they do it and how it works. I can also observe the fine nuances that can be between a healthy part and a survival part. And the intention, because a representative can quickly shift depending on where the client is, between being in survival or a healthy part” (O)*.

### To Witness and Be Witnessed on Multiple Levels in a Safe Space

To see the self and the effects of trauma on the self under guidance of a therapist are central to the resonance process and journey toward self-discovery, as stated from all perspectives. Being witnessed, by the therapist and potentially representatives and observers, also holds a special power. From the client perspective, being seen is an empowering acknowledgment. The seeing part entails a sense of recognition, a process wherein the body may recognize the truth, which in optimal conditions occurs within a safe and non-judgmental space. “*//the feeling of being understood, the feeling of being seen, the feeling that something is finally being recognized, these feelings. The feeling of like ‘Oh, finally.' // Acknowledged” (C)*. Through IoPT work, the client gets to see parts of the self and their interaction played out on the floor as the representatives make them visible when resonating with the client's sentence of intention. Or the client gets to feel the parts if resonating with the markers him-/herself. The parts visualized through resonance refer to the ternary model, including the healthy, traumatized and survival parts of the split human psyche. The witnessing through resonance of the experienced trauma and its consequences, survival strategies and healthy parts, helps understand reaction patterns. It can spread light on the origin of survival strategies and their past value, but also their potentially hindering effects long-term. Past experiences—whether conscious or not, previously known or not, or stemming from the self or previous generations—and their consequences for the client's identity development become visible. “*The advantages with this method is that I can get hold of things I wasn't aware of, didn't have contact with, laying in the unconscious, pushed down in the body throughout life. So the opportunity to see it, and hear the representatives narrate things that resonate with me, that kind of ‘pling, aha, yes that's true, I had forgotten”' (C)*. Although painful, this process can be liberating, healing and self-developing, and thus helpful. “*So there have been some really painful…. acknowledgements of biography that I don't have in recall but my body knows. So painful and helpful” (C)*. The visualization process helps the client discern and acknowledge the truth as experienced by him/her. It provides an explanation to the state of things and possibly an aha-experience in which things fall into place and make sense. “*//seeing through the resonance, and you can't ignore it because it is in your face. And it hurts ‘Oh, this is what I am doing towards myself or towards others.' Really to see it outside yourself in your resonance//” (C)*. The process may also be difficult to grasp, or find echo in, and result in more questions than answers. The client's ownership of his/her truth and biography are nevertheless central premises, and only (s)he knows whether the process resonates with him/her, and if/how it makes sense. The facilitator can check in with the client by asking if and how what is shown in the process resonates with him/her.

Central to IoPT work is the emotional contact, also felt physically through bodily sensations, that can be made with the parts of the self that were split off through trauma. Participants narrate that in comparison to other modalities, IoPT is less cognitive and reaches deeply and quickly to the roots of trauma, without lengthy talk, even though the process of integration may take time. “*But you reach so much deeper so much quicker with IoPT. // With IoPT you get contact with the things you don't want to talk about, can't talk about, or don't know how to talk about. You get help to talk about it with the help of your representatives. You get help to come into contact with it” (T)*. Acceptance of the self as it is shown in the process, through recognition or acknowledgment, enables processing and integration of the self through acceptance of the shown parts as parts of the self. “*Acceptance comes before you can integrate” (R)*. Altogether, this contributes to a cognitive understanding of trauma and its consequences, and compassion toward the self, e.g., as painful experiences and subsequent reactions are acknowledged and accepted, and possibly embraced with compassion. It helps discern the past from the present and to let go of the past. The process thus involves contact on multiple levels, i.e., emotional, physical and cognitive. “*That's the great key. Because you can't understand this emotionally, physically and cognitively at the same time with any other method. // It's the seeing that becomes an understanding, which becomes a letting go. It's a happening in the moment of seeing, understanding, feeling, compassion” (C)*.

The need or wish to verify what comes up in the process against external facts can vary. Some facts are known and/or recognized when shown, not requiring external verification. While some facts cannot be verified, others are verified *via* external sources (e.g., witnesses, documents). Varied reasons may refrain verification, e.g., a reluctance to consult witnesses or expectations of denial. With references made to verification needs being a survival strategy, focus is however brought back to the centrality of knowing and owning one's own truth, how it was experienced by the self. This minimizes needs for verification with facts or others' perceptions. “*I also need to find out what happened then…. Maybe it's important, but no, I don't know. // Sometimes, I've needed to, not always. It's actually what it did to me that is becoming more important” (C)*.

### The Subject in the Driver Seat

Specific to IoPT as narrated by the participants is its' self-regulating and client driven approach, experienced as a unique and challenging strength. This is a central and guiding principle in IoPT work from all perspectives, whether acting in the client, therapist, representative or observer role. “*Here the therapist is a guide and the client is in the driver seat and steers” (T)*. Without facilitator intervention, the subject sets his/her intention. Further (s)he chooses representatives (if available, i.e., in group sessions), supporting autonomy and self-responsibility. It contrasts the sense of helplessness and lack of control associated with trauma. “*The main thing is the way of facilitating, which is none intrusive, so held back. So respectful of the autonomy of the client, even if the client himself does not think he has any autonomy at all” (T)*. The client raises issues that (s)he is ready to face, presupposing motivation and a “state of readiness,” which guides the intention setting process in a self-regulating manner. Clients cannot order specific answers through the process; issues at stake will show in the process, whether the client can or cannot take them in for various reasons, is open or not to what the resonance shows (e.g., not ready, in denial, lack of recognition/resonance in the client). If central, key issues will reoccur in future processes. “*If things come up that you're not ready to take in, you just won't see them. If you can't take them in, it's like ‘I think that was a bit strange.' It's completely ok. Then you just bring it with you and it will come up when it's time. It's a beautiful, inbuilt support” (C)*. The facilitator shall not force anything upon the client and must respect if the client wants to end the process. The facilitator may ask whether and how what is shown in the process resonates with the client, and can offer the client a description and interpretation of what (s)he observes based on his/her knowledge and experience of IoPT, if the client wants to hear it. In dialogue with the facilitator, the client can steer the sessions' frequency and pace. From the client and facilitator perspectives, the client driven approach and more equally leveled client-facilitator status reduce risks of manipulation and retraumatization, not the least when contrasted to modalities with more “paternalistic or authoritarian” approaches. “*I know that my therapists have worked with this // I know that my supervisor also does that. It's not just a technique. Then we're also more on the same level. It's not just me as a client, it's not just the therapist that is elevated, we're on the same level” (C)*. Yet the potential value of complementary modalities is acknowledged, although opinions, knowledge and experience levels diverge on this point. Combinations with additional supportive modalities may be viewed as legitimate and necessary, or as hindering or slowing down IoPT work.

### Navigation Through Facilitation, With or Without Crew

While the client is in the driver seat, the therapist, or facilitator is steadily present as a guide in IoPT processes, with an overview of the client, representatives, observers, and the dynamics in the room. This requires a number of conditions from the facilitator. Facilitation is a balancing act that requires awareness, presence, clarity, and openness in the facilitator, in combination with solid knowledge and experience. The facilitator helps with navigation and meaning making throughout the process; nevertheless, only the client is the true holder of his/her experiences. “*I am not here to tell you about your own life, only you know that, you and your psyche have been there the whole time. So you and your psyche know how much you want to explore. My role is to provide you with a safe vehicle” (T)*. Compassion and non-judgment contribute to a safe space. Confidence in the facilitator role, the client and his/her autonomy, the representatives, trust in the process and a safe framework based on ethical conduct and confidentiality principles are fundamental for effective processes. The facilitator must be able to “hold and stay with the process,” i.e., contain the process. (S)he must be comfortable with not having answers nor solving the client's issues, and allow the process to unfold without taking ownership of it or jumping to (potentially faulty) conclusions. “*Always realize yourself that I as facilitator can't have a solution, can't find a solution” (T)*. Observation of the resonance process informs the narrative and potential inquiries through which the facilitator can guide the client between the resonance points. (S)he can offer hypotheses based on observations of the process and on theoretical, methodological and experiential knowledge of IoPT at a pace that is bearable to the client. Nor too pushy, at risk of retraumatization and dissociation in the client, nor too withdrawn. “*One must really stand not to know// where it all goes. One may see, recognize after a while, but hold // not to know, and still be present and not freak out and start looking for explanations. Keep it open //Otherwise it becomes a false memory. Very important” (T)*. Facilitator availability post-process is desirable. It permits questions and reflection about the process. “*//there are some times when I've had questions, or clarification questions that I might speak with the facilitator about, or email the facilitator. And certainly I also do that with clients, ‘if something comes up after the session and you want to ask questions” (C/T)*.

Shortcomings in the above-mentioned areas may represent a risk to client safety, and decrease the client's trust in the process and facilitator. “*[the facilitator] would not leave enough time for the process to develop naturally// and she would put words too quickly in the mouths of the representatives, or she would give explanations too quickly. I don't find that helpful at all. It twists the process, it manipulates the process. And then I started to not trust anymore” (C)*. Facilitation presupposes own IoPT work as a client and regular supervision, addressing facilitator challenges and client cases. “*As I clear up my stuff, I become a better and better navigator. // You resonate better and better, you resonate deeper and deeper.// I think if you really as a facilitator do the training, do the work, read the books, get involved with the theory, if you really do the work, it is pretty clear…. There is a pretty clear path to not being a perpetrator” (T)*. Dealing with one's own trauma biography is necessary to hold the space, as is respect of one's comfort zone, to reduce risks of triggers, facilitation from blind spots, or manipulation of the process. Own work in combination with the client-steered process are seen as risk reducing in terms of potential manipulation and retraumatization, also compared to other modalities. The relatively frequent occurrence in clients' processes of sexual abuse is reflected upon from multiple perspectives: is it due to its actual high frequency, to IoPT representing a particularly suitable and empowering modality to address such issues, and/or to other factors? “*Because it happens so often, many children are sexually abused by people whom they should be able to trust.//it is just happening behind many front doors, more than we know” (T). “Maybe I'm a bit oversensitive about the sexual abuses // I have some question marks there sometimes and then…// it's a matter of interpretation, what is… // then one must define what sexual abuse is. //within the method, there may be a danger as a therapist when it comes to finding a balance between what is the truth and what is a proposal, and what is the… actual interpretation… of what is being shown, so to say. // I'd say that the risk lays more in the individual therapist rather than… but of course, it becomes even more sensitive maybe when one is dealing with areas // that are difficult to verify against an external reality” (C)*.

The facilitation process also depends on crew size as group and one-to-one facilitation (OTO), with representatives and/or markers, offer different prerequisites to the process. IoPT facilitators can work with only one or several formats, depending on preferences and resources, e.g., in form of representatives. Clients may also choose depending on preferences if both formats are available. Both OTO and group processes add value to the client's self-discovery and development process, and can be used alone or in combination, face-to-face or *via* online video chat. Assets in group processes are the externalized visualization of, interaction and dynamics between the parts in the process, and potentially the superior amount of information shown through the representatives as compared to OTO sessions. “*Whereas the advantage in group is absolutely that all your basic dynamics that are mirrored by the resonance points are active simultaneously” (C)*. In OTO sessions with markers, the double facilitator role entails a challenge and need for pedagogical clarity, but also comprehension from the client as the facilitator moves from one marker to another, consecutively resonating with the different markers and acting as both facilitator and resonator. “*The patient must be able to understand that now it's the therapist ‘entering' me, then it's the therapist speaking, and then the therapist sits down on the chair. So it demands something of the patient, that (s)he is not too unwell” (T)*. OTO sessions may be more private, less intimidating and limited information wise, thus potentially less overwhelming and easier to overview. Nevertheless, the interaction and dynamics between the parts will not be visualized as in a group process with the parts simultaneously present. In OTO sessions where the client him-/herself moves from one marker to the other, s(he) will experience the differences in resonance on the different markers, hence him-/herself embodying the different parts of the self through resonance. The client's experiential work becomes tangible. “*Certainly in a one-to-one session, when the client comes off one resonance and goes into another, they can't deny that there is a change. You know, they're in one resonance and they come off. And they set into the next resonance and there is immediate – they say ‘oh my god, I feel sick.' I mean, that demonstrates the resonance process” (T)*.

### Meaning of the Crew

The group can be an asset on several levels from all perspectives. It naturally affects the facilitation format with a group setup, with the possibility to overview and follow the dynamics and interaction between the different parts enacted by the representatives, as compared to OTO processes. A group is a source of representatives, which has its intrinsic reciprocal value as participants stand by each other in their respective processes as clients. It can be a circle of trust, and offer an opportunity for participants marked by unreliable and harmful relationships to experience new, trustful ways of relating with others. “*You get a feeling of great support, as there are several persons you know that take care of you. For me who don't trust people// I start to do that now, but I am terrified of people because my closest ones abused me. // Now I don't feel lonely anymore.// So the whole session widens my perspective on what people can be for me” (C)*. Group participants are the client's witnesses, an important aspect as seen earlier. They can be a source of support through their mere presence, compassionate attitude and shared experiences, contributing to holding the space. “*Apart from experiences that you're not alone, that you have witnesses and that there is a holding power, which is very, very good” (C/T)*. Being a witness to others' experiences contributes to an increased understanding of and compassion toward fellow humans. These aspects presuppose trust in the group, which is essential for prosperous processes and to which the facilitator can contribute by establishing a trustful framework. A group setting can also be intimidating, and OTO processes therefore an option. Being a representative, and thus part of the group, also has its inherent value in a person's journey toward (self) exploration and discovery (see below).

### Resonance as a Phenomenon and Role

The presence of and access to representatives is tangible from all perspectives, whether as a client, therapist, representative or observer. It affects the IoPT processes' format and those involved in the process. The subject of resonance comes through as a phenomenon through which the self is made visible for the client, an essential IoPT ingredient as seen in the witnessing process. It also comes through as a role through which the representative tangibly experiences the client's internal world. “*Somehow words, feelings, physical sensations, acts, movements come by themselves.// Being a representative has been the clearest for me because I really feel that ‘this isn't me,' and still I feel and say all this. But now I can show all these things// sometimes one is chosen… because it's an nearby issue. Just as I know that it's only the other one that has moved in, I still know and feel that it also affects my own issues. I can separate the two. And then it is healing” (R)*. The act of resonance may be one of reciprocity as participants offer to be each others' representatives. It can add value also to the representative as (s)he comes into contact with a variety of emotions and sensations, whether previously familiar or not to him/her. The “by-pass” as a resonance point can help open up for and explore such emotions. The resonance process may touch upon adjacent issues although not necessarily, and stimulate (self) reflection in the representative. It can thus contribute to an enhanced understanding of, respect for and compassion toward the self and others from multiple perspectives.

Separating own issues from the client's when resonating, and trust in the representative from the client perspective, are key. It appears to be relatively straightforward, although doubts may arise in both clients and representatives. Verbalizing one's doubts as a representative in connection with the process can help address such issues. The facilitator can also check in with the client to hear how the process resonates with him/her. “*If I'm insecure as to whether it's mine or the client's, I can remark on it loudly when I say it, like ‘I'm not sure whether this belongs to me or to the client, but I say it”' (R)*. Similarly to clarity in the facilitator, clarity in the representatives contributes to increased trust in the process from the client's perspective. Experiences as a representative also enhance the understanding of and trust in the method and representative role, especially when representatives see that the client recognizes their resonance. “*How strange it can be to know nothing about the person and yet manifest something which seems to be spot on for this person. This is so amazing” (R)*. Trust further expands when recognition occurs in processes where the client and representatives are unknown to each other. “*And that for me was a break-through in understanding what was being manifest was what was going on for me, but also trusting in the phenomena. There is no way that woman could have known that sentence!” (C)*. Difficulties exiting the representative role after the process occasionally occur. Hypotheses relate to the process being deep, from early stages of life, or intense for the client, but also to the process having touched upon issues that activate the representative's own triggers and identifications with the resonated parts. If the client repeats the process of letting go, it generally solves the issue. “*It has happened a few times, when I needed to be taken out twice. But I think it was when something was close to my own issues” (R)*. Patterns in resonance roles one is chosen to resonate with may or may not be observed, and changes over time may be interpreted as a sign of self-development by the person in the representative role.

### Reclaiming the Self

IoPT work offers a self-explorative journey that contributes to an enhanced self-knowledge and development. Clients and therapists equally work with own IoPT processes as clients, representing an opportunity for self-development. In some cases, representatives can be part of that journey, with or without observers, and also learn from their participation in IoPT processes. Landing in one's own identity through IoPT work paves a way for the self to develop and “own” its space. “*I see different cards from different stages in my history, in my biography. And that I can see, this is not about here and now, these cards are here for me now still, but that it is from there and then, and I have a choice. It's a big difference” (C)*. Separating the past from the present becomes easier once acknowledging the traumatized (child) parts, paving way for a more adult and autonomous stance. “*What's in the driving seat is the survival strategy of the child. And the decision making capacity of the adult is a very different place to live in than the survival strategy of the child. Of my child, my childhood state” (C)*. Rather than identification with others, the self is the new point of reference, also affecting relations with e.g., enhanced relationships and the release of detrimental ones. Identifying old triggers through the witnessing and resonance processes helps adjust behavioral patterns to better serve the self. Again, participants refer to the traumatized, survival and healthy parts, as described in the ternary model. “*You can help the client separate the past from the present. Because clients are often triggered in the present. The triggers give bodily sensations, bodily pain or anxiety, or whatever. Once they learn that what happens in the present is a reaction to what happened in the past and how you can deal with it, and how you can recognize your traumatized part and how your survival parts take over. You make a definite separation between the two, and you can learn that you can use your healthy parts to become stronger” (T)*.

With consideration taken to difficulties pinpointing cause-effect or confounding factors, central changes are still credited to IoPT work. The latter facilitates changes and a depth of work never reached before, e.g., through other therapeutic modalities or restorative processes. “*It has been a profound shift in my life, I mean profound. // I have tried one thing after the other to somehow clarify what is troubling me. Nothing came close, not even close, to what this did within two sessions” (C)*. An internal pre-process build-up, including the intention setting process, can be followed by a post-process release, sometimes with subsequent symptoms such as tiredness and sickness but also heightened energy. Physical symptoms and medication intake may diminish or disappear over time. Other noticeable changes for instance include feeling calmer, less anxious or worried, more open, grounded, self-confident, self-accepting, compassionate and self-responsible. “*After these constellations, I got in touch with my own feelings, which I've been keeping down my whole life. I feel quieter, more settled.// I'm more in the comfort zone” (C)*. Having acknowledged the truth, one can now rest with this knowledge. “*I understood my life and I understood myself better. Once you understand yourself and you know what has happened, you feel ‘Oh well…' It sort of brings some lightness and some more quietness in your being” (C)*. Changes include listening to one's needs and body, looking after oneself and setting healthy limits, rather than focusing others' needs or expecting others to take care of one's needs. Energy previously employed by survival strategies can be redirected at approaches that are more helpful. “*I experience that when I take responsibility for my needs, the relation // doesn't have to take responsibility for my needs” (C)*.

### The Black Box—Struggling With an Explanation and Pedagogical Model

IoPT reaches early and deep levels of trauma when compared to other modalities, contributing to its effects. How to explain the processes and mechanisms at work in IoPT nevertheless arouses deliberations in the participants, regardless of perspective. Recurrent references are made to IoPT theory and method, and to the significance of the intention setting process and e.g., mirror neurons in the resonance process. Mentions are made of scientific areas such as e.g., neurosciences, epigenetics and quantum theory. “*I have found including the neuroscience into my psychoeducation with clients as a useful way for me to explain to clients what is happening and what these processing sessions offer. I am very intrigued about how other people do it. Because there is no form of training in how you do that. //We're on a training program, but what it is, is experiential sessions so everybody has their own processing session as a way of deepening your understanding of trauma from this model. It doesn't have anything about therapeutic protocol” (T)*. Unquestionable though is the sense of amazement at the spot on character of IoPT work and its effects. A healthy skepticism, as contrasted to blind faith, parallels trust in theory and method, which is prompted by firsthand experience of IoPT. “*One shall always have a little sound skepticism towards all kinds of therapy, as there are good psychologists and not so good psychologists, good psychiatrists and not so good psychiatrists. //So far I'm not that critical because as long as it benefits me, why should I be skeptical?” (C)*. The theory seems more easily introduced and accepted in new settings than the method as it is yet relatively unknown, barely scientifically researched despite the clinical experience, and sometimes difficult to explain. For the uninitiated, the trauma concept may be scary and foreign. “*I'd like to start my own business, but over the past 2½* *years with IoPT I feel that it's a challenge to convey it to people that have never tried it.// we're afraid of that phenomena [fundamentalism], that something is to be the solution, and I absolutely agree with that fear. At the same time, I can't disregard IoPT's uniqueness. I think its a big 'dilemma' (T)*. One contemplates ways to integrate IoPT with own values and work models while staying true to the original IoPT modality. The variation in terms of one's previous therapeutic experiences as a client, one's professional education and occupation (including of psychotherapeutic nature), may be a factor at play. “*It's important that the therapist is confident with the method and confident with how (s)he presents him/herself and the method. // One needs to pack this, to present it so elegantly that this magic - I call it magic - that arises when we manage to read each other, it should be described in a way that results in ‘Yes, I understand that's how the method works”' (T)*.

## Discussion

### Results

The current study sheds light on experiences of IoPT from multiple perspectives. This may be a strength, as the findings point to the value and merging of multiples roles, and a limitation as it limits the specificity and depth of understanding of individual perspectives. Through the constant comparative method, a tentative theory came to show the significance of the intertwined perspectives in relation to the participants' experiences of self-discovery and self-development through IoPT. Further studies may focus the separate perspectives to deepen our understanding of their unique characteristics. The examination of a topic from multiple perspectives can nevertheless contribute to a more complete understanding of the depth and breadth of human experiences (72). Witnessing the self as a client and being witnessed come through as key aspects in IoPT processes, where the multiple perspectives also can be at play. The intention setting method and resonance process have central roles in this process. The value of getting to see and face one's subjective truth, of getting an explanation to one's experiences, is empowering and liberating although painful. The witnessing part illuminates one of several dimensions of the group's significance, for instance in renegotiating the meaning and potential value of relationships. The findings corroborate the value of social support and of sharing similar experiences at times of distress, which can reduce feelings of loneliness and alienation (73–75). This may be particularly valuable for persons with a trauma history, marked by feelings such as sadness, distrust, helplessness, guilt and shame (46).

The current findings highlight the importance of the facilitator's capacity to harbor emotions, hold a safe space, and explore the process together with the client in an empathic and non-judgmental way. The therapeutic alliance is a well-known variable that contributes to the effectiveness of psychotherapies (76), including assets such as emotional responsivity and empathy (77). The current study's participants contrast the more equal status of facilitator and client in IoPT to more traditional psychotherapeutic modalities, experienced as more authoritarian. Aspects of IoPT that come through as central are its client-driven and so-called self-regulating approach, in the sense that only what the client is ready to address will drive the process and affect what (s)he is ready to see, accept and integrate through the process. Focus is on client autonomy and self-responsibility, and on the client's ownership of his/her process, trauma biography and truth. The facilitator has a central balancing role. These aspects, in combination with the therapist's own IoPT processes and supervision, were deemed to minimize risks of manipulation and retraumatization, also when compared to other modalities. Potential risks are inherent to any therapeutic modality and practitioner. The findings show that competence, experience, a safe framework and trust were essential to secure client safety, as was facilitator availability. The therapist must be able to contain the process and safely guide the client. Safety of experiential work within a therapeutic relationship can help the client first feel and then reflect upon difficult affects, while working with empathy-based, affect-regulating and attuned relatedness (78). Working with emotions, emotional regulation, and meaning making are common ingredients in trauma-oriented psychotherapies (46). Germer and Neff (79) further point to the value of self-compassion in trauma treatment, both as a resource in the therapeutic relationship and as a quality to cultivate in the client, which participants also pinpointed in the current study. Self-kindness, common humanity and mindful awareness can strengthen the client's capacity to tolerate and transform traumatic memories through safe exposure and non-avoidance. It can soothe internal reactions to stress, such as self-criticism, self-isolation, self-absorption (79). The current study did not specifically explore the issue of regulating strategies in IoPT work, but participants described IoPT as a self-regulating approach. This may be of interest to explore in more depth considering their importance in trauma treatment (46, 48, 52, 53). It goes in line with paying attention to the client's “window of tolerance” (80) or “optimum arousal zone” (44), especially as traumatized individuals may have poor tolerance for arousal (81).

As described by the participants, the experienced contact on both emotional and physical levels through resonance were central in IoPT processes, also contributing to a cognitive understanding in the client. This goes in line with the fact that emotional experiences are not processed through language and logic (78), as in talk therapies (82). They ought to be addressed in a right hemisphere language, i.e., sensations, images, impressions, and urges toward action (78), of which the activation can make visible implicit trauma based emotional memories (82). Access to sensory, motoric, and somatic experiences, as in experiential therapies with bottom-up approaches, facilitates the processing of emotionally traumatic experiences and a dyadic affect regulation process (83). Signs of dissociation and survival mechanisms can be illuminated as conscious and unconscious emotional reactions, body-based emotional information, and non-verbal communication are visualized (41), including perceptions, sensations, emotions, thoughts and intentions (68). The body and associated sensations may nevertheless be a scary place for persons with a trauma history, which is why the therapist must be aware of the client's limits and state of readiness to confront traumatic memories. IoPT facilitation entails a balancing role in present awareness, including re-orientation of the client to the present if needed. Participants highlighted the experiential and less mental nature of IoPT, which reaches rapidly and deeply to the origins of trauma without the need for lengthy talk, especially when compared to other therapeutic modalities. As Gerge (48) states, talk therapies may be more suited for persons without complex trauma, as talking about trauma may reactivate instead of heal trauma. Signs of dissociation and complex trauma must be observed or patients will not get better and patients need help with strategies to handle affects that were split when trauma occurred (48). Working with memories, but also with coping skills and psychoeducation are further commonalities in therapies addressing trauma (46). Good knowledge of psychotraumatology is thus necessary as unready patients may risk retraumatization when opening-up for unintegrated material. Therapists can mirror the client and model affect-regulative strategies, especially as these parts may have been missing in the parent-child interactions (78). The workings and value of IoPT as narrated in the current study go in line with experientially natured therapeutic modalities. As seen, the facilitator has a balancing role. S(he) must tolerate to hold the process, safely guide the client through the process, and be present and observant of potential retraumatization and dissociation risks.

Participants struggled with explanatory and pedagogical models for IoPT. They made recurrent references to IoPT theory and method, and to the significance of e.g., neurosciences. There was an element of wonder about the mechanisms at work, which seemed difficult to grasp and express in words for the participants, although work with IoPT processes was experienced as tangible in its effects. The resonance process is central to IoPT work, motivating further research on the subject. The findings in the current study cannot answer to the mechanisms at work or causational relations, only tentatively shed light on the participants' subjective experiences of IoPT. IoPT leans against traumatology and psychological research, including the importance of e.g., attachment theory, mirroring and mentalisation (54–58), which resonates well with the literature (77, 84). It also goes in line with the idea of revisiting traumatic memories to integrate them, of making visible parts of the self that developed defensive habits to survive, and with the notion of restoring a sense of agency and ownership of body and mind in the client (46, 84). Ruppert advocates that IoPT can help clients handle different types of early trauma and offer insight into inner conflicts. This can help strengthen healthy parts of the personality, visualize survival strategies, and discover more constructive ways of relating to reality, which increases the client's level of freedom and well-being (55); assertions on a par with the current findings.

### Strengths and Limitations

The method was inspired by grounded theory, which is well-suited for unexplored areas. The researcher's understanding and experience of IoPT developed throughout the study, enabling both a naïve outlook denuded of preconceived ideas and the development of theoretical sensitivity along the research process. Own experiences from the international training further contributed to firsthand experiences and an enhanced theoretical and practical understanding of IoPT. The researcher's observational and interpretive skills and experiences, along with data interactions throughout the study, helped inform the emerging theory (69, 72). The regular and parallel use of a diary and memos were used to support reflexivity (60). Only one researcher carried out the study, limiting the possibility for interrater-reliability. Nevertheless, comparison itself works as a verifier of data in grounded theory, grounding the emerging substantive theory in data (60). The researcher made sure to revisit emerging themes and categories throughout the interviews and to work with the constant comparative method to enhance the findings' reliability. Quotes were included for transparency of the analysis process. Trustworthiness refers to the theory's fit to the substantive area and its relevance for the participants (61). The theory must work to explain relevant behavior in the substantive area and be readily modifiable as new data emerges, possibly enriching the theory (61). Further studies can thus enhance our understanding of IoPT and its value, and potentially corroborate or expand on the current findings by illuminating further variability (71).

The sample was self-selected, limited in size and may not be representative of a wider population. Its characteristics in terms background variables may have affected the findings and limit transferability of the results. Nonetheless, the study was explorative in nature, opening up for the generation of hypotheses and further studies on the subject. Despite the limited sample, the individual interviews and focus groups generated rich data and a sense of saturation. One can however not guarantee that further data collection will not generate new data. This motivates further studies with the inclusion of larger samples with variations in terms of sociodemographic factors and with specific focus on exploring the separate perspectives in more depth. In the current study, only two participants solely had experiences from the client perspective, which may have affected the current findings. A more detailed description of the participants' therapeutic training in other schools of psychotherapy is lacking, although such experiences were touched upon during the interviews, showing that such experiences were common. This could however be looked upon in more depth in future studies. The semi-structured guide helped cover multiple aspects associated with IoPT, contributing to a homogenous data collection and the coverage the explored perspectives across all interviews. The suitability of such a guide with grounded theory can be discussed. Nonetheless, all participants were invited to speak freely and there was room for flexibility to explore areas of interest in more depth in the forthcoming interviews, based on previous interviews. No causal inferences can be made as the study was explorative and qualitative in nature. It shed light on significant transformative experiences in the participants, which can be further explored in future studies.

The in-depths interviews shed light on profound changes in the participants' life, view on themselves, and relationships through their experiences of IoPT. Psychotherapy research that mainly focuses effects or process factors that contribute to change come with methodological challenges (76, 77). This may be especially valid when studying IoPT as it has developed over time, potentially affecting dependability and replicability of the results if the method further develops. Braud and Anderson (72) state that research on human experiences that are “*personal, subjective, significant*, and *relevant*” (p. 19) may require alternative research approaches as such experiences may be inwardly experiential and subjective. They can be significant and transformative in the informant's and others' life, but may be kept private and are hence difficult to assess by external observers at the *time of occurrence* (72). The current study illuminates tangible transformative experiences in the participants, which they also reflected upon throughout the interviews. The researcher also witnessed such processes during the training modules, further informing her theoretical sensitivity and understanding of IoPT. Such transformative experiences may help the person “*rediscover, remember, and relive aspects of being and of self that had been forgotten, ignored, or neglected*” [(72), p. 20]. The latter goes in line with the findings that IoPT work facilitates the discovery of the inner self and access to recondite dimensions of the self. Besides their transformative implications on individual and group levels, such experiences may affect social, political, and ethical dimensions (72). Ruppert's (85) pursued work points that way. Increased focus on the meaning of interrelational stress may contribute to a systemic viewpoint on mental illness and its treatment (86). As research can be value laden (72), consideration of the researcher's and the researched phenomenon's cultural and historical contexts may be motivated. Further research on systemic levels may hence be motivated.

## Conclusion

The current study sheds light on experiences of IoPT from multiple perspectives. It shows the significance of the intertwined perspectives in relation to the participants' experiences of self-discovery and self-development. While the examination of a topic from multiple perspectives can contribute to a more complete understanding of the depth and breadth of human experiences, it may limit an in-depth understanding of the separate perspectives. Further, the study highlights the importance of the facilitator's capacity to harbor emotions, hold a safe space, and explore the process together with the client in an empathic and non-judgmental way. The findings show that competence, experience, a safe framework and trust were essential to secure client safety, as was facilitator availability. The facilitators' capacity, education and competence thus come through as key aspects at play that need consideration in IoPT work to achieve beneficial effects and prevent potential re-traumatisation. The deleterious effects on health of prolonged stress and emotional and physiological hyperarousal, and the enhanced exposure to risk factors and risky health behaviors subsequent to early and recurrent trauma exposure, generate substantial individual suffering and costs to society. As trauma affects the nervous system with effects on body, mind and brain, it is of relevance for trauma treatment to work with strategies that engage the whole organism on multiple levels (84). Addressing traumatic experiences and renegotiating relationships to the self and others through a safe therapeutic relationship can help clients restore a sense of agency, security and hope, and help them engage in more healthy behaviors and more fully in life. The prevention of and recovery from ill health through effective treatment modalities contribute to beneficial effects on individual, group, and societal levels. The limited evidence in relation to effective treatments of complex trauma motivates the study of novel approaches such as IoPT, including digital treatment formats (49). As seen in the current findings, both face-to-face and digital formats are practicable with IoPT.

Due to the paucity of scientific publications on IoPT, an explorative, qualitative research approach was chosen for the current study, with its inherent strengths and limitations. This single researcher study limits the possibility for interrater-reliability. The sample was self-selected and may not be representative of a wider population. Whether the self-selected sample includes participants that are positively biased toward IoPT cannot be answered. The researcher was nevertheless consistent in posing questions tapping into both positive and negative aspects of the participants' experiences with IoPT. The sample's characteristics in terms background variables may have affected the findings and limit transferability of the findings. Further studies with larger and varied samples are thus motivated to expand on the current study's findings. In terms of strengths, the chosen explorative approach was useful. The author's own experiences from the international training further contributed to firsthand materials and to a deeper understanding of IoPT that developed throughout this empirical study. Furthermore, the individual interviews and focus groups generated rich data, covering multiple aspects associated with IoPT, which can be explored in more depth in future studies. The current study nonetheless illuminates the value and transformative potential of IoPT from multiple perspectives, including experienced strengths and risks associated with IoPT work. Further qualitative and quantitative research may enhance our understanding of the mechanisms and value of IoPT to promote self-development, health and quality of life.

## Data Availability Statement

The datasets generated for this study will not be made publicly available for ethical reasons, not to compromise anonymity or confidentiality agreements.

## Ethics Statement

The study was approved by the Regional Ethical Committee in Lund, Sweden (reg.nr 2018/921). All participants signed a written, informed consent prior to the interviews and agreed on the publication of quotes in the current article.

## Author Contributions

SS planned and carried out all stages of the current study's research process.

## Conflict of Interest

The author declares that the research was conducted in the absence of any commercial or financial relationships that could be construed as a potential conflict of interest.
