# Supplementary material for: Getting to Know the Inner Self. Exploratory Study of Identity Oriented Psychotrauma Therapy—Experiences and Value From Multiple Perspectives
Source: Front Psychiatry. 2021 May 21;12:526399. doi: 10.3389/fpsyt.2021.526399 (PMC8175788; doi:10.3389/fpsyt.2021.526399)
Supplement: Supplementary file 1 [file Data_Sheet_1.PDF]

Supplementary data

Appendix 1: Interview guide

I. Client Perspective, with questions (6-13) inspired by Elliotts' Change Interview (1996)

1. How did you first come into contact with IoPT?
2. What motivated you to try IoPT?
3. Did you get help to address the issues/reasons that motivated you to try IoPT?
4. What are your previous experiences of therapy, if any? Are you are willing to share these shortly?
5. What are the advantages/disadvantages of IoPT compared to your previous therapeutic experiences?
6. General experience of therapy: What has IoPT been like for you (so far)? How has it felt to be doing IoPT work?
7. Changes: How are you doing now? What changes, if any, have you noticed in yourself since you started with IoPT?
8. Attributions: In general, what do you attribute these various changes to? What do you think might have brought them about (outside/inside IoPT)?
9. Helpful aspects: What have been the most helpful things about IoPT so far? (general aspects/specific events) What made these things helpful to you?
10. Hindering aspects: were there aspects of IoPT that you experienced as hindering, unhelpful, negative or disappointing?
11. Difficult but OK aspects: Were there things in IoPT work that were difficult or painful, but still OK or helpful?
12. Missing aspects: Was there anything missing in the IoPT modality?
13. Suggestions: Do you have any suggestions regarding IoPT?
14. Is there anything that I have not asked about or something that we have not talked about from the client perspective that you want to share?

II. Therapist perspective

1. Which therapeutic orientation do you identify with, if any? How do you combine IoPT with your other orientation, if any?
2. Which indications/counter-indications do you see for IoPT as a therapeutic method?
3. What kind of issues do clients wish to solve through IoPT, according to your experience?
4. Which processes (within/outside therapy) and mechanisms (within therapy) do you think are at work within IoPT that result in changes in the client?
5. Which (unique) advantages and strengths do you see with IoPT compared to other types of therapy?
6. Do you see any particular disadvantages and risks with IoPT compared to other types of therapy?
7. Which intensity of therapy do you recommend?
8. Which, if any, difficulties do you experience as a therapist when using IoPT? How do you handle them?
9. What are your experiences of supervision?
10. Can IoPT replace other therapeutic interventions or should IoPT be considered as a complement?

15. Is there anything that I have not asked about or something that we have not talked about from the therapist perspective that you want to share?

III. Representative perspective

1. Which are your experiences of being a representative in another person's constellation?
2. Can you explain what happens?
3. Do you experience any particular difficulties or challenges in the role as a representative?
4. Do you experience any personal value from the role as representative in other people's constellations? If so, can you explain what, how, in which way?
5. Can you easily separate what belongs to the client and what belongs to you (during/after the constellation)? How do you handle that?
6. Have you ever said no to being a representative when chosen? Do you want to say why?
7. Is it difficult to get out of the role as a representative? What do you need to be released?
8. Is there anything that I have not asked about or something that we have not talked about from the representative perspective that you want to share?

IV. Observer perspective

1. In which context were you an observer?
2. What are your experiences of being an observer? (thoughts, feelings, etc)
3. Can you see/experience any value from being an observer? If so, can you please describe that value?
4. Is there anything that I have not asked about or something that we have not talked about from the observer perspective that you want to share?
